# The bovine leukemia virus-derived long non-coding RNA AS1-S binds to bovine hnRNPM and alters the interaction between hnRNPM and host mRNAs

**DOI:** 10.1128/spectrum.00855-23

**Published:** 2023-09-06

**Authors:** Kiyohiko Andoh, Asami Nishimori, Yuichi Matsuura

**Affiliations:** 1 National Institute of Animal Health, National Agriculture and Food Research Organization, Tsukuba, Ibaraki, Japan; University of Florida College of Dentistry, Gainesville, Florida, USA

**Keywords:** AS1-S, bovine leukemia virus, hnRNPM, lymphoma, non-coding RNA

## Abstract

Viruses utilize several strategies to cause latent infection and evade host immune responses. Long non-coding RNA (lncRNA), a class of non-protein-encoding RNA that regulates various cellular functions by interacting with RNA-binding proteins, plays important roles for viral latency in several viruses, such as herpesviruses and retroviruses, due to its lack of antigenicity. Bovine leukemia virus (BLV), which belongs to the family Retroviridae, encodes the BLV-derived lncRNA AS1-S, which is a major transcript expressed in latently infected cells. We herein identified bovine heterogeneous nuclear ribonucleoprotein M (hnRNPM), an RNA-binding protein located in the nucleus, as the binding partner of AS1-S using an RNA-protein pull-down assay. The pull-down assay using recombinant hnRNPM mutants showed that RNA recognition motifs (RRMs) 1 and 2, located in the N-terminal region of bovine hnRNPM, were responsible for the binding to AS1-S. Furthermore, RNA immunoprecipitation (RIP) assay results showed that the expression of AS1-S increased the number of mRNAs that co-immunoprecipitated with bovine hnRNPM in MDBK cells. These results suggested that AS1-S could alter the interaction between hnRNPM and host mRNAs, potentially interfering with cellular functions during the initial phase of mRNA maturation in the nucleus. Since most of the identified mRNAs that exhibited increased binding to hnRNPM were correlated with the KEGG term “Pathways in cancer,” AS1-S might affect the proliferation and expansion of BLV-infected cells and contribute to tumor progression.

**IMPORTANCE:**

BLV infects bovine B cells and causes malignant lymphoma, a disease that greatly affects the livestock industry. Due to its low incidence and long latent period, the molecular mechanisms underlying the progression of lymphoma remain enigmatic. Several non-coding RNAs (ncRNAs), such as miRNA and lncRNA, have recently been discovered in the BLV genome, and the relationship between BLV pathogenesis and these ncRNAs is attracting attention. However, most of the molecular functions of these transcripts remain unidentified. To the best of our knowledge, this is the first report describing a molecular function for the BLV-derived lncRNA AS1-S. The findings reported herein reveal a novel mechanism underlying BLV pathogenesis that could provide important insights for not only BLV research but also comparative studies of retroviruses.

## INTRODUCTION

Viruses utilize several strategies to maintain latent infection while evading host immune responses (1). One of the strategies is the expression of functional non-coding RNA (ncRNA); the viral genome encodes ncRNA to modulate the gene expression of host or viral genes in infected cells (2 – 5). Since ncRNAs can evade host immune responses due to their lack of antigenicity, they are an effective tool for maintaining latent infection. A representative example of ncRNA is miRNA, which is a class of approximately 21 nucleotide small RNAs that repress gene expression by increasing RNA degradation or inhibiting translation, thereby altering numerous cellular processes in infected cells (6). Another ncRNA is long non-coding RNA (lncRNA), a class of RNAs with lengths of >200 nucleotides that do not encode protein; most lncRNAs regulate various cellular functions by interacting with the genome and RNA-binding proteins (3, 4, 7).

Bovine leukemia virus (BLV), which belongs to the genus Deltaretrovirus of the family Retroviridae and causes malignant B cell lymphoma in cattle, encodes several ncRNAs, including miRNA and lncRNA (8 – 11). BLV expresses structural and accessory proteins and aggressively expands during the early stage of infection. However, upon establishing a latent state during chronic infection in cells, BLV expresses few viral antigens encoded in the sense strand of the genome (12, 13). In contrast to the protein-coding transcripts, expression of miRNAs and antisense transcripts, all of which are lncRNA, continues during latent infection (9, 11, 14). Among these ncRNAs, the miRNAs are likely responsible for BLV pathogenesis (15, 16). The BLV-derived lncRNA AS1 consists of two isoforms, AS1-L and AS1-S, and is expressed in the nucleus of BLV-infected cells (11, 14). Although the function of AS1-S remains unknown, it is hypothesized to play important role in the BLV lifecycle due to its continuous expression.

Since most lncRNAs function by interacting with RNA-binding proteins, AS1-S potentially interacts with several proteins in infected cells, and the interactions between AS1-S and its binding partners might play pivotal roles in modulating the cellular environment during latent infection or in the progression of lymphoma. Therefore, identification of the binding partners of AS1-S and clarification of its function would enable elucidation of the biological function of BLV-derived lncRNAs. In this study, we aimed to identify the binding partners of AS1-S RNA and to comprehensively explore the phenotypic changes brought by the interactions between them. We identified bovine heterogeneous nuclear ribonucleoprotein M (hnRNPM), an RNA-binding protein located in the nucleus, as the binding partner of AS1-S. hnRNPM is involved in several cellular processes, such as mRNA splicing and the formation of subnuclear structures, which possibly affect the BLV lifecycle from various aspects (17, 18). The AS1-S-expressing cells showed changes in the variety of mRNAs that co-immunoprecipitated with bovine hnRNPM, indicating that AS1-S modified cellular functions by altering the interactions between hnRNPM and host mRNAs in the nucleus.

## RESULTS

### Construction and evaluation of AS1-S RNA-expressing Madin-Darby bovine kidney (MDBK) cells

To evaluate the functional changes associated with AS1-S in the cells, we established MDBK cells expressing AS1-S under the control of the CAG promoter or its internal 3´ long terminal repeat (LTR) promoter (Fig. S1A through C in the supplemental materials). The relative amount of AS1-S RNA in the nucleus and cytoplasm of these cells was measured using real-time reverse transcriptase (RT)-PCR, and the results indicated that the relative amount of AS1-S in the nucleus of transgenic MDBK cells was smaller than that of the BLV-infected B cell line BL3.1. AS1-S in BL3.1 cells was mainly located in the nucleus (approximately 70%) while the transgenic AS1-S in MDBK cells was mainly located in the cytoplasm (<30%) (Fig. S1D in the supplemental materials). Notably, the amount of nuclear AS1-S RNA in MDBK CAG AS1-S cells was quite low (<10%) relative to that in the MDBK 3´LTR AS1-S cells (approximately 30%), which was consistent with a previous report indicating that an antisense RNA driven by a strong promoter was localized to the cytoplasm (19). The relative amounts of the nuclear [U6 small nuclear RNA (U6)] and cytoplasmic [tyrosinase (TYR)] RNA controls in the transfected cells were consistent with those in BL3.1 cells, supporting the validity of the experiment (Fig. S1D in the supplemental materials). These results indicated that MDBK 3´LTR AS1-S cells were more suitable than MDBK CAG AS1-S cells for use in the subsequent experiments, although the relative amount of nuclear AS1-S RNA was lower in MDBK 3´LTR AS1-S cells than in BL3.1 cells.

### Transcriptome analysis of transfected MDBK cells

**Fig 1:**
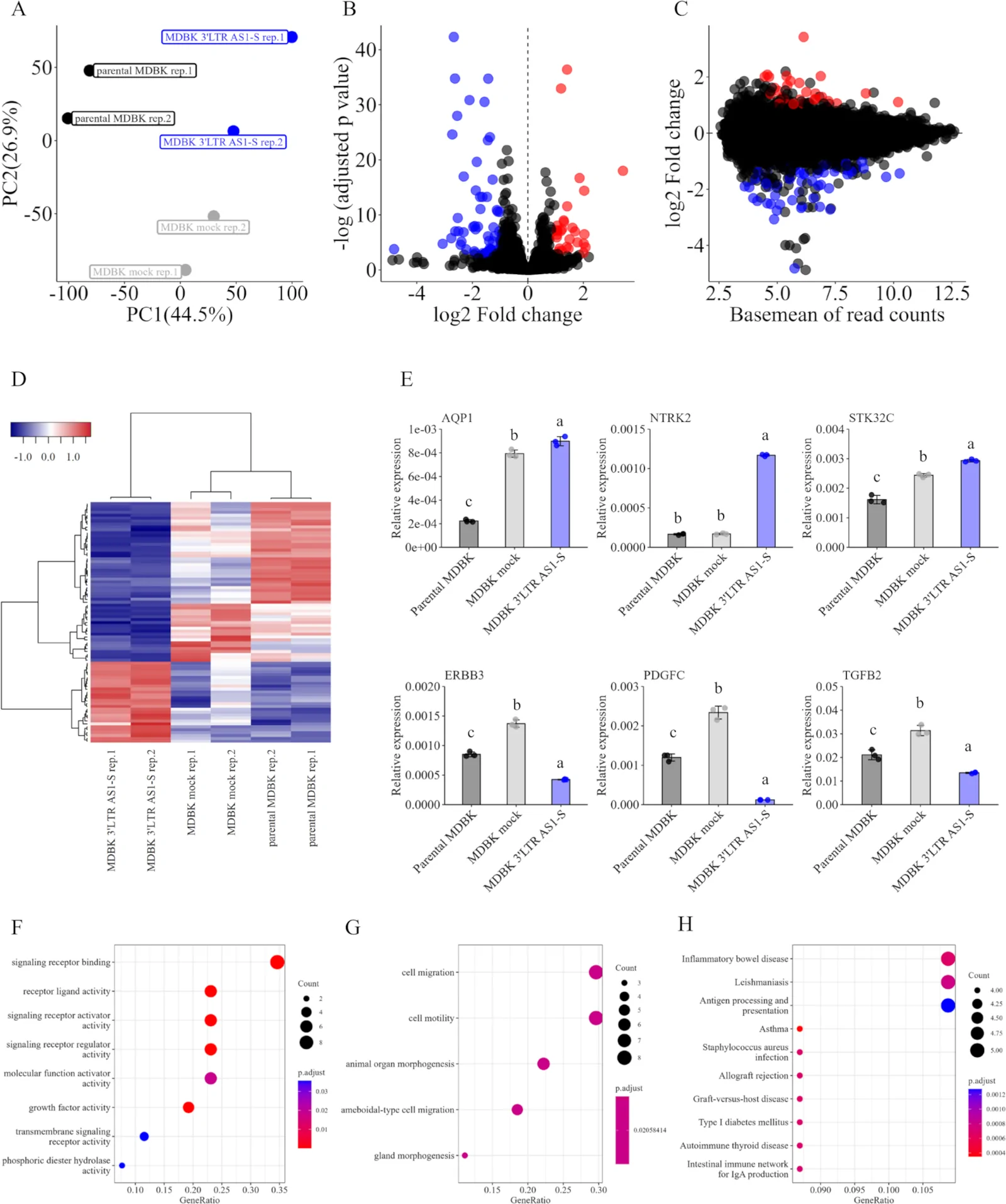
(**A**) Principal component analysis plot of the transcriptome analysis using MDBK 3´LTR AS1-S, MDBK mock, and parental MDBK cells. The X and Y axes show principal components 1 and 2, and percentages in parentheses indicate their respective contributions to the overall variability. Volcano plot (**B**), smear plot (**C**), and heatmap (**D**) of the transcriptome analysis results. Significant differentially expressed genes (DEGs) identified between the MDBK 3´LTR AS1-S cells (*n* = 2) and the MDBK mock and parental cells (*n* = 4) are shown in red and blue (up- and downregulated in MDBK 3´LTR AS1-S cells, respectively). Significant DEGs were defined based on the following criteria: |fold change| ≥2 and exactTest adjusted *P*-value < 0.05. (**E**) Quantification of mRNAs in MDBK 3´LTR AS1-S, MDBK mock, and parental MDBK cells; six genes identified as DEGs were subjected to validation using real-time RT-PCR. The results are shown as the expression levels relative to *GAPDH*. Data are presented as the mean ± standard deviation (*n* = 3), and different letters indicate statistically significant differences at *P* < 0.05 with Tukey’s test. (F–H) Gene ontology (GO) analysis of significant DEGs. The enriched terms in molecular function (MF) (**F**) and biological process (BP) (**G**) and the KEGG pathway enrichment analysis (**H**) are shown separately. The size of the bubbles indicates the gene count, and the color of the bubbles indicates the adjusted *P*-value. An adjusted *P*-value < 0.05 was defined as statistically significant.

The transfected MDBK cells were subjected to transcriptome analysis in an effort to identify differentially expressed genes (DEGs). A principal component analysis (PCA) plot showed that MDBK 3´LTR AS1-S cells were clustered separately from the mock and parental cells. However, MDBK mock cells also exhibited an altered expression profile when compared to the parental MDBK cells due to insertion of the empty vector or antibiotic selection (Fig. 1A). Subsequently, the mRNA expression profile of MDBK 3´LTR AS1-S cells (*n* = 2) was compared to those of the MDBK mock and parental cells (*n* = 4) to identify AS1-S-specific effects. The resultant differential expression analysis identified 83 DEGs in MDBK 3´LTR AS1-S cells, with 28 upregulated and 55 downregulated genes (Fig. 1B through D, the DEGs are listed in Table S2 in the supplemental materials). Six of the 83 DEGs were validated using real-time RT-PCR, and the results confirmed that the upregulated (*AQP1*, *NTRK2*, and *STK32C*) and downregulated (*ERBB3*, *PDGFC*, and *TGFB2*) DEGs in MDBK 3´LTR AS1-S cells were expressed the most and the least, respectively, with statistical significance, in the three groups, which was consistent with the results of the transcriptome analysis (Fig. 1E).

The identified DEGs were then subjected to gene ontology (GO) analysis, and the results showed that the molecular function (MF) terms “signaling receptor binding,” “receptor ligand activity,” “signaling receptor activator activity,” and “signaling receptor regulator activity,” and the biological process (BP) terms “cell migration,” “cell motility,” and “animal organ morphogenesis” were significantly enriched (Fig. 1F and G, the results of the GO analysis are listed in Table S3 in the supplemental materials). Moreover, KEGG pathway enrichment analysis showed that the terms “Inflammatory bowel disease,” “Leishmaniasis,” and “Antigen processing and presentation” were enriched (Fig. 1H, the results of the KEGG pathway enrichment analysis are listed in Table S4 in the supplemental materials). Multiple comparison analyses also showed that terms related to signaling pathways were significantly enriched in AS1-S-expressing cells, which was consistent with the results from the two-group comparisons (Fig. S2; Tables S5 and S6 in the supplemental materials).

### Identification of a host-derived protein that binds to the AS1-S RNA probe

**Fig 2:**
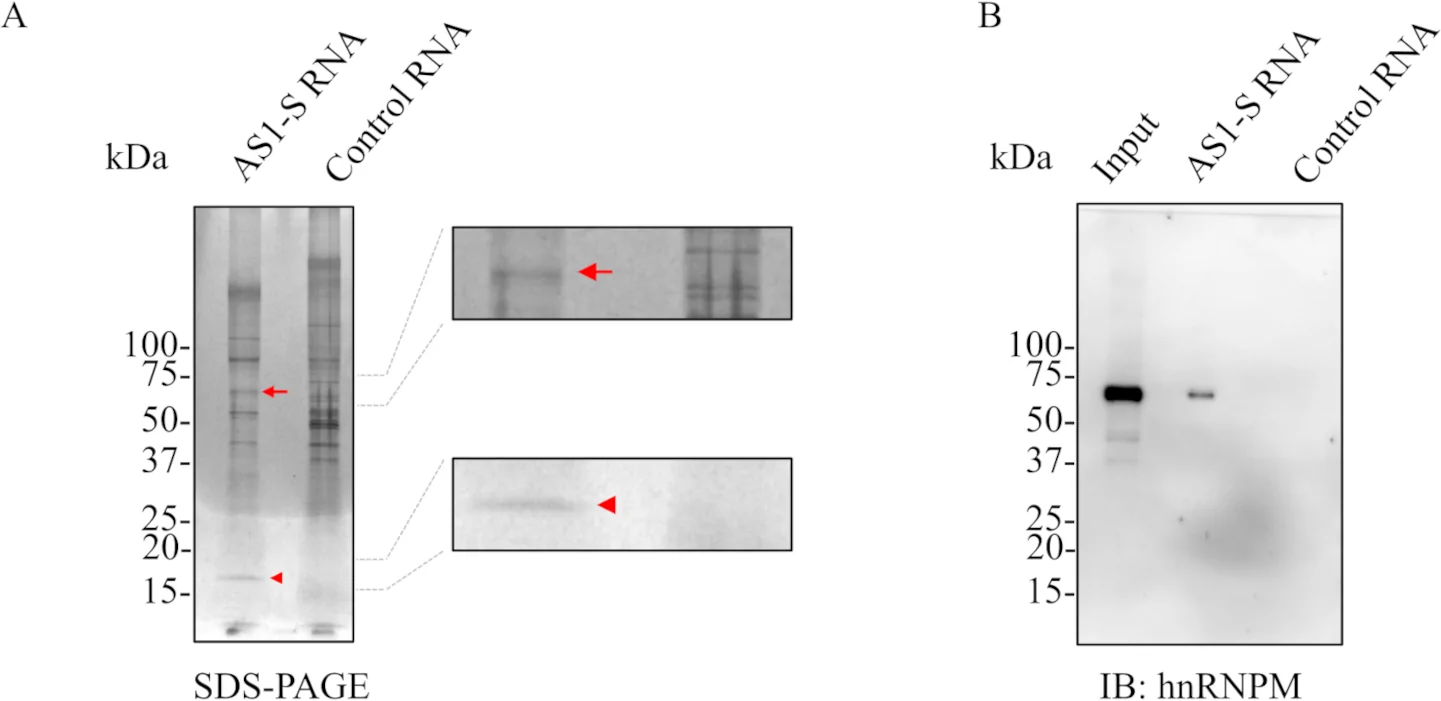
Results of the pull-down assay using RNA probes. Biotinylated AS1-S or control RNA probes were mixed with a BL3.1 cell lysate, and pulled-down samples were subjected to SDS-PAGE (silver staining) (**A**), followed by western blotting (**B**). The red arrow shows the band that was predicted to be bovine hnRNPM by LC-MS. The arrowhead shows the band that is specific for the AS1-S RNA probe but did not produce a significant result with LC-MS.

Since the expression of AS1-S altered gene expression in MDBK cells, we attempted to identify the binding partner of AS1-S using an AS1-S RNA probe and BL3.1 cell lysates. The RNA-protein pull-down assay results revealed two bands that were specific to the sample obtained with the AS1-S RNA probe; the sizes of the bands were approximately 70 kDa and <20 kDa (Fig. 2A; Fig. S3 in the supplemental materials). The two bands were analyzed by liquid chromatography-mass spectrometry (LC-MS), and the approximately 70 kDa band was predicted to be bovine hnRNPM. To confirm the result obtained by LC-MS, western blotting analysis using an anti-hnRNPM monoclonal antibody was performed, and the results showed a band specific for bovine hnRNPM at approximately 70 kDa in the sample from the AS1-S RNA probe (Fig. 2B). These results indicated that AS1-S RNA physically interacted with bovine hnRNPM.

### Confirmation of the interaction between AS1-S and hnRNPM in bovine B cells

**Fig 3:**
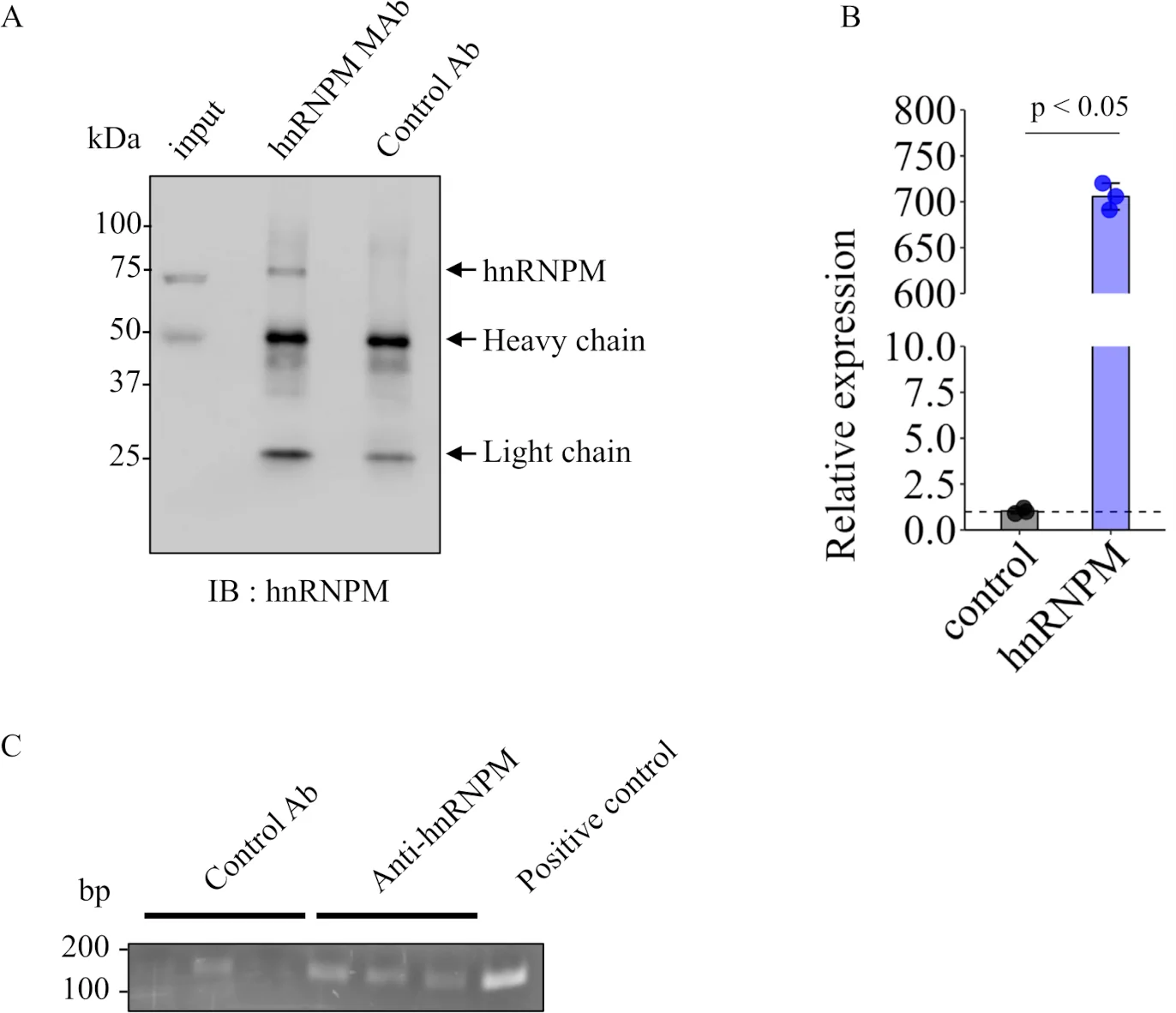
(**A**) Western blotting analysis of RNA-immunoprecipitated samples. RNA-protein complexes were immunoprecipitated from BL3.1 cell lysates using an anti-hnRNPM or control monoclonal antibody and then subjected to western blotting. Heavy and light chains observed in the immunoprecipitated samples were derived from the antibodies used for the immunoprecipitation procedure. (**B**) Quantification of AS1-S RNA in the RNA-immunoprecipitated samples from BL3.1 cells. RNA was purified from the RNA-protein complexes obtained by RNA immunoprecipitation, followed by real-time RT-PCR. The results are shown as the expression levels relative to the control sample. Data are presented as the mean ± standard deviation (*n* = 3), and a *t*-test was performed for statistical analysis. *P*-value< 0.05 was defined as statistically significant. (**C**) PCR amplification of AS1-S RNA in RNA-immunoprecipitated samples from primary bovine lymphocytes. RNA purified from the RNA-protein complexes obtained by RNA immunoprecipitation was subjected to real-time RT-PCR, followed by electrophoresis. The positive control is a PCR amplicon obtained from the RNA-immunoprecipitated sample of BL3.1 cells.

To confirm whether the interaction between AS1-S and bovine hnRNPM occurs in BLV-infected B cells, an hnRNPM-RNA complex was extracted from BL3.1 cells using an RNA immunoprecipitation (RIP) assay. The RIP assay results indicated that the anti-hnRNPM antibody specifically immunoprecipitated bovine hnRNPM (Fig. 3A), and real-time RT-PCR showed that the amount of AS1-S RNA that immunoprecipitated with anti-hnRNPM was approximately 700 times greater than the amount that immunoprecipitated with the control antibody (Fig. 3B).

Next, to confirm whether the interaction between AS1-S and bovine hnRNPM occurs in primary cells *in vivo*, bovine primary lymphocytes were subjected to the RIP assay. Primary lymphocytes were obtained from the lymph node of a necropsied BLV-positive calf, and real-time PCR for measuring the BLV proviral load showed that 17.6% of the cells were BLV-positive. The results from the RIP assay and subsequent real-time RT-PCR revealed that AS1-S RNA was detected in the sample from RIP with the anti-hnRNPM antibody (mean Ct value = 33.71 ± 0.35) but not that with the control antibody; since the relative amount could not be calculated from real-time RT-PCR, the PCR amplicons were directly confirmed by electrophoresis (Fig. 3C). The results suggested that AS1-S RNA interacted with hnRNPM in BLV-infected B cells.

### Identification of bovine hnRNPM regions responsible for binding AS1-S

**Fig 4:**
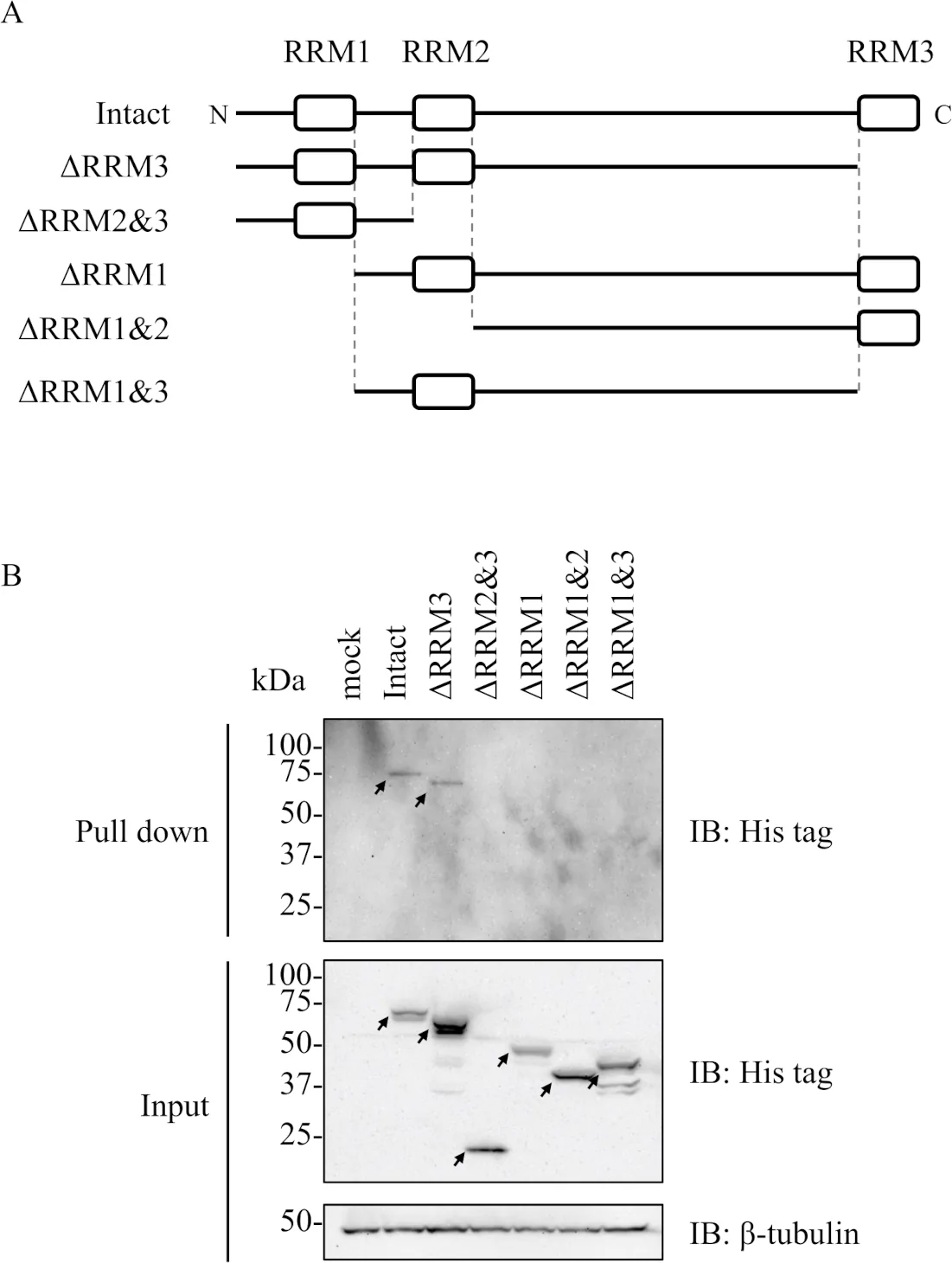
(**A**) Schematic diagrams of the bovine hnRNPM protein and the constructed deletion mutants. White boxes indicate the RNA recognition motifs (RRMs). All constructs were fused with a his-tag sequence and inserted into the expression plasmid pCAG neo. (**B**) Results of the pull-down assay using the AS1-S RNA probe and recombinant hnRNPMs. Recombinant proteins were expressed in 293T cells, and the obtained cell lysates were mixed with a biotinylated AS1-S RNA probe and subsequently assessed by western blotting. Arrows indicate the predicted sizes of the recombinant proteins.

Bovine hnRNPM contains three RNA recognition motifs (RRMs) in its amino acid sequence (20). To identify the regions responsible for binding AS1-S, bovine hnRNPM deletion mutants were constructed and subjected to the pull-down assay (the mutant constructs are shown in Fig. 4A). The results of the pull-down assay using the mutant constructs and the AS1-S RNA probe showed that only the full-length and ΔRRM3 hnRNPMs, both of which include RRM1 and RRM2, are bound to the AS1-S RNA probe (Fig. 4B). This indicated that both RRM1 and RRM2 were required for the interaction between bovine hnRNPM and AS1-S.

### Knockdown of hnRNPM expression in BL3.1 cells

To confirm whether knockdown of hnRNPM affects the expression of viral proteins in BL3.1 cells, siRNAs targeting bovine hnRNPM were transfected into BL3.1 cells. Western blotting analysis results showed that the expression of hnRNPM was reduced by the transfection of two siRNAs targeting hnRNPM (sihnRNPM #1 and #2). However, the expression of viral proteins, such as gp51 and p24, was not affected in these cells (Fig. S4).

### Comprehensive analysis of hnRNPM-binding RNAs in transfected cells

**Fig 5:**
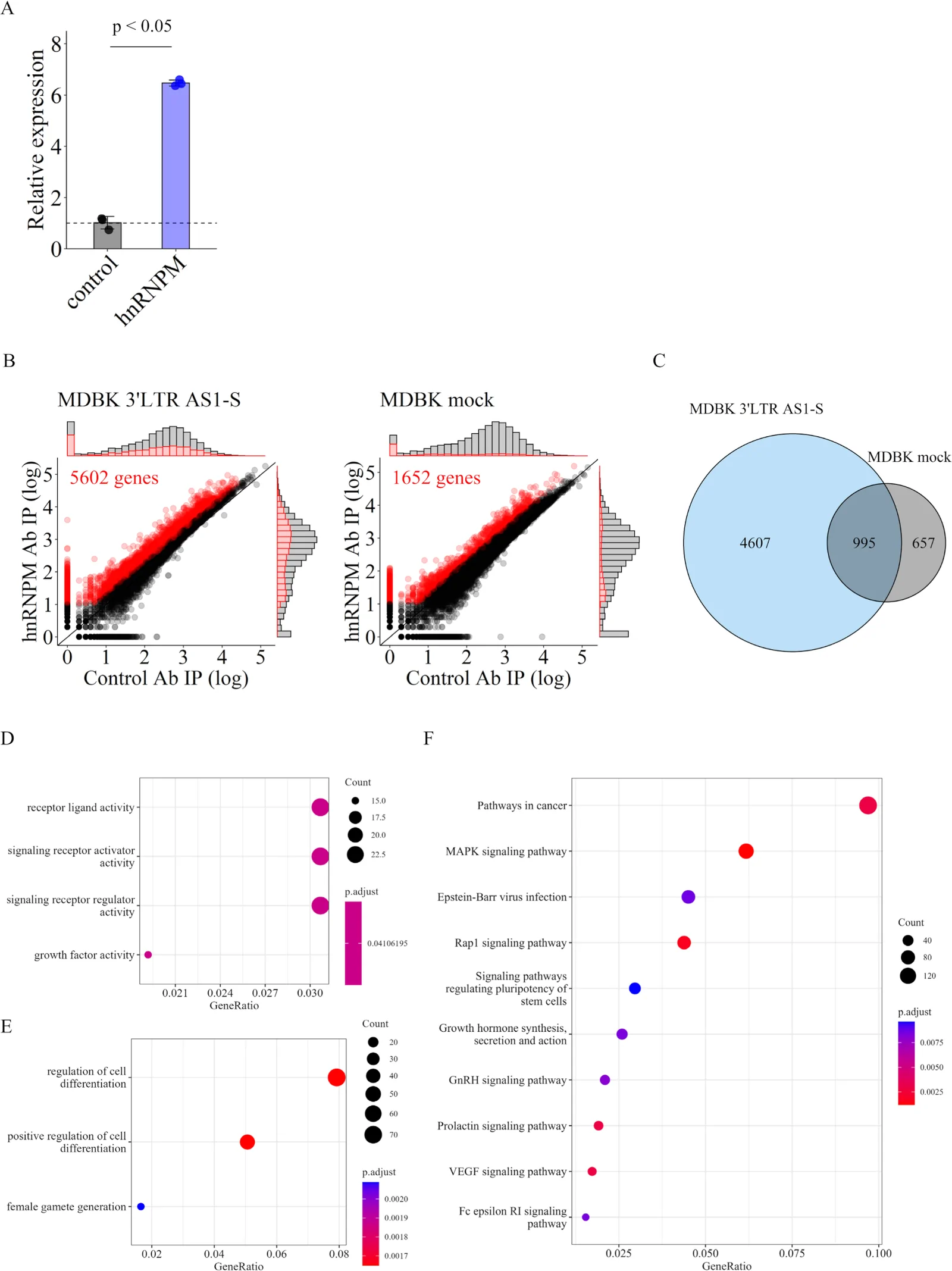
(**A**) Quantification of AS1-S RNA in RNA-immunoprecipitated samples from MDBK 3´LTR AS1-S cells. RNA was purified from the RNA-protein complexes obtained by RNA immunoprecipitation, followed by real-time RT-PCR. The results are shown as the expression levels relative to the control sample. Data are presented as the mean ± standard deviation (*n* = 3), and a *t*-test was performed for statistical analysis. *P* < 0.05 was defined as statistically significant. (**B**) Scatter plots showing the sequencing reads obtained by RNA immunoprecipitation. The X and Y axes show the read counts obtained by RIP-seq with the control MAb and anti-hnRNPM MAb, respectively. Genes that met the following criteria were defined as hnRNPM-binding RNAs and are shown as red dots: a read count >10, hnRNPM/control ratio >2.0. (**C**) Venn diagram showing the overlapping hnRNPM-binding genes in MDBK 3´LTR AS1-S and MDBK mock cells. (D–F) Gene ontology (GO) analysis of the 4,607 hnRNPM-binding genes detected only in MDBK 3´LTR AS1-S cells. The enriched terms in molecular function (MF) (**D**) and biological process (BP) (**E**), and the KEGG pathway enrichment analysis (**F**) are shown separately. The size of the bubbles indicates the gene count, and the color of the bubbles indicates the adjusted *P*-value. An adjusted *P*-value < 0.05 was defined as significant.

Because AS1-S interacted with the RRMs in hnRNPM, it is possible that the interaction between AS1-S and hnRNPM affects the interactions between hnRNPM and endogenous RNAs. To evaluate whether AS1-S interferes with the interactions between hnRNPM and host-derived RNAs, the RNAs interacting with hnRNPM were comprehensively analyzed by RIP-seq. The results of the RIP assay using the transgenic MDBK cells showed that the amount of AS1-S RNA that immunoprecipitated with the anti-hnRNPM antibody was approximately six times greater than that with the control antibody in MDBK 3´LTR AS1-S cells (Fig. 5A), indicating that exogenous AS1-S bound to hnRNPM in MDBK cells. The RIP samples were subsequently subjected to RNA-seq analysis (RIP-seq), followed by visualization as read counts for each mRNA (Fig. 5B). The results showed that the number of mRNAs that co-immunoprecipitated with hnRNPM (a read count >10, hnRNPM/control ratio >2.0) in MDBK 3´LTR AS1-S and MDBK mock cells was 5,602 and 1,652, respectively (shown as red dots in Fig. 5B, the RIP-seq results are shown in Tables S7 and S8 in the supplemental materials). Analysis of the gene list revealed that 995 genes were observed in both MDBK 3´LTR AS1-S and MDBK mock cells, and 4,607 of 5,602 genes (82.2%) were observed only in MDBK 3´LTR AS1-S cells (Fig. 5C). This result suggested that the expression of AS1-S increased the variety of mRNAs that co-immunoprecipitated with hnRNPM.

To identify common functions between the 4,607 mRNAs identified in Fig. 5C, the gene list was subjected to GO analysis. The result indicated that the MF terms “receptor ligand activity,” “signaling receptor activator activity,” and “signaling receptor regulator activity” as well as the BP terms “regulation of cell differentiation” and “positive regulation of cell differentiation” were significantly enriched (Fig. 5D and E, results of enrichment analysis are listed in Table S9 in the supplemental materials). Furthermore, KEGG pathway enrichment analysis showed that most of the identified genes were related to pathways for cell proliferation, such as “Pathways in cancer,” “MAPK signaling pathway,” “Epstein-Barr virus infection,” and “Rap1 signaling pathway” (Fig. 5F; Fig. S5, results of the enrichment analysis are listed in Table S10 in the supplemental materials).

**Fig 6:**
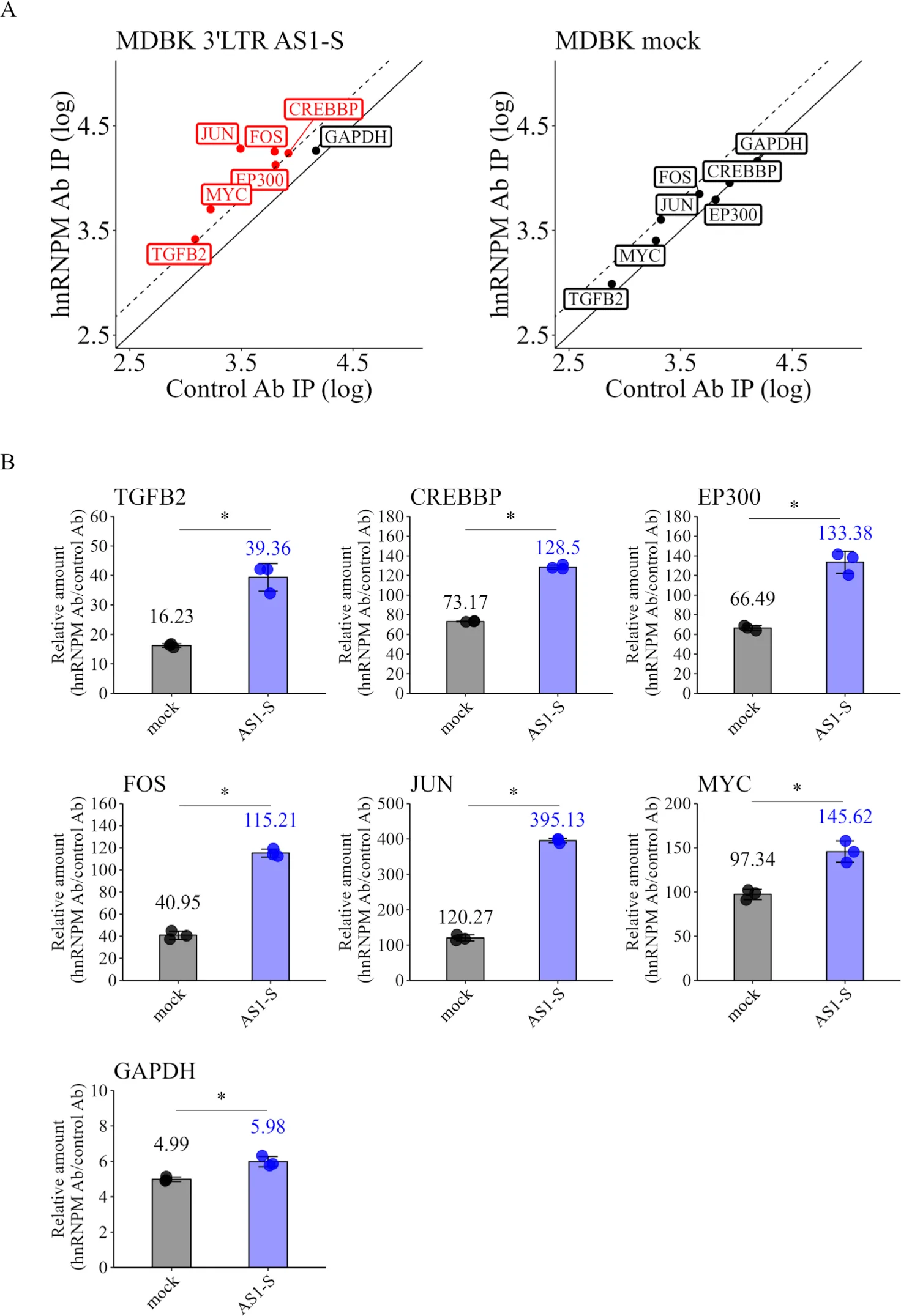
(**A**) Scatter plots showing the sequencing reads obtained by RNA immunoprecipitation; seven representative genes subjected to real-time RT-PCR are shown. The X and Y axes show the read counts obtained by RIP-seq with the control MAb and anti-hnRNPM MAb, respectively. Genes that met the following criterion are shown as red dots: hnRNPM/control ratio >2.0. (**B**) Quantification of seven mRNAs in the RNA-immunoprecipitated samples from MDBK 3´LTR AS1-S and MDBK mock cells. RNA was purified from the RNA-protein complexes obtained by RNA immunoprecipitation, followed by real-time RT-PCR. The results are shown as the amounts of RNA in the sample from RIP with the anti-hnRNPM antibody relative to that with the control antibody. Data are presented as the mean ± standard deviation (*n* = 3), and a *t*-test was performed for statistical analysis. *P-value* < 0.05 was defined as statistically significant. The mean values are shown at the top of the bars.

To validate the results of RIP-seq, the amounts of the RNAs in the RIP samples obtained from MDBK mock and MDBK 3´LTR AS1-S cells were determined using real-time RT-PCR; six genes included in the 4,607 genes in Fig. 5C and the GO term “Pathways in cancer” were selected, and *GAPDH* was also selected as a control gene (Fig. 6A). The validation results showed that the amount of RNA in the sample from the RIP with anti-hnRNPM antibody relative to that with control antibody was significantly higher in MDBK 3´LTR AS1-S cells than in MDBK mock cells; the fold changes in *TGFB2*, *CREBBP*, *EP300*, *FOS*, *JUN*, and *MYC* (2.42, 1.76, 2.01, 2.81, 3.29, and 1.50, respectively) were higher than those in *GAPDH* (1.20) (Fig. 6B).

### Analysis of the mRNA distribution in AS1-S-expressing cells

Since several heterogeneous nuclear ribonucleoproteins are involved in the nuclear export of mRNAs (21, 22), we measured the nuclear and cytoplasmic RNAs in MDBK 3´LTR AS1-S and MDBK mock cells and comprehensively evaluated the distribution of the RNAs to confirm whether the interaction between AS1-S and hnRNPM affected mRNA translocation. The read counts of the nuclear and cytoplasmic RNAs showed that the nuclear/cytoplasmic RNA ratio was similar in AS1-S-transfected and mock cells (Fig. S6A, the count matrix is shown in Table S11 in the supplemental materials). Moreover, the 4,607 mRNAs identified in Fig. 5C exhibited a similar nuclear/cytoplasmic RNA ratio in AS1-S-transfected and mock cells (Fig. S6B in the supplemental materials). These results indicated that the expression of AS1-S did not affect the nuclear export of mRNAs.

### Analysis of the mRNA distribution and hnRNPM-binding RNAs in BL3.1 cells

We also analyzed the mRNA distribution and hnRNPM-binding RNAs in BL3.1 cells to obtain supplemental data, although no proper control, such as BLV-negative B cells, could be prepared. The scatter plots of the nuclear and cytoplasmic RNAs in BL3.1 were largely identical to those in MDBK 3´LTR AS1-S and mock cells (Fig. S7A, the count matrix is shown in Table S12 in the supplemental materials). In contrast, the RIP-seq results differed remarkably from those of MDBK-derived cells; the number and amount of RNAs that co-immunoprecipitated with the anti-hnRNPM were greater than those that co-immunoprecipitated with the control antibody in BL3.1 cells (Fig. S7B, the RIP-seq results are listed in Table S13 in the supplemental materials). Western blotting analysis showed that the expression level of hnRNPM was almost equal among the samples with the same number of BL3.1 and MDBK-derived cells (Fig. S7C in the supplemental materials), although the expression of beta-tubulin in BL3.1 cells was less than that in MDBK cells. This result indicated that the differences observed in the RIP-seq might be attributable to the cell type or BLV infection.

## DISCUSSION

In this study, we identified bovine hnRNPM as the binding partner of the BLV-derived lncRNA, AS1-S. Pull-down assays using recombinant hnRNPMs showed that both RRM1 and 2 were responsible for binding to AS1-S (Fig. 3). In addition, we found that the expression of AS1-S increased the variety of RNAs that co-immunoprecipitated with bovine hnRNPM in MDBK cells. Moreover, the number of mRNAs that formed a complex with hnRNPM in AS1-S-transfected cells was approximately three times greater than that in mock cells, and KEGG pathway enrichment analysis results suggested that most of the identified RNAs were related to the KEGG term “Pathways in cancer” (Fig. 5). These results indicated that AS1-S may alter the interactions between hnRNPM and mRNAs and potentially affect the proliferation and expansion of infected cells, which could be novel mechanisms for the progression of lymphoma. To the best of our knowledge, this is the first report attributing molecular functions to BLV lncRNA.

Since AS1-S is bound to the RRM1 and 2 of hnRNPM, we hypothesized that AS1-S might physically interfere with interactions between hnRNPM and host RNAs. Contrary to our expectation, the expression of AS1-S increased the number of mRNAs co-immunoprecipitating with hnRNPM in MDBK cells (Fig. 5B and C). As a consequence, AS1-S was hypothesized to alter the interactions between hnRNPM and host mRNAs, resulting in the regulation of the expression of several genes during the initial transcription processes that occur in the nucleus. Although it remains unknown how AS1-S RNA increases the interactions between hnRNPM and several mRNAs and whether the identified mRNAs directly bind to hnRNPM, liquid-liquid phase separation (LLPS) could be implemented to explain the mechanism. Previously, Unfried and Ulitsky proposed that lncRNAs were able to form biomolecular condensates in cells and that the biomolecular condensates could facilitate enzyme activities by locally increasing the concentration of enzymes and substrates (23). Their review suggested that such reversible condensates may compensate for the low expression levels of lncRNAs (23). Therefore, AS1-S RNA could function in the same manner to form a condensate with hnRNPM, thereby altering the hnRNPM-RNA interactions. Moreover, the present RNA-protein pull-down assay data suggested that the AS1-S RNA probe could bind hnRNPM as well as other host-derived proteins (Fig. 2). Thus, it is possible that several unknown molecules also support the formation of biomolecular condensates and increase interactions between hnRNPM and mRNAs. This hypothesis is consistent with a previous report demonstrating that AS1-S RNA was observed as small nuclear dots by *in situ* hybridization (14).

In the present study, we could not identify specific genes or pathways that were directly affected by the interaction between AS1-S and hnRNPM since numerous factors were identified by RIP-seq and KEGG pathway enrichment analysis (Fig. 5; Fig. S5 in the supplemental materials). However, these analyses showed that the KEGG pathway “Pathways in cancer” was significantly enriched (Fig. 5F), indicating a high possibility that AS1-S acts as a determinant for the abnormal proliferation of BLV-infected cells. Generally, pathways related to cancer primarily facilitate cell proliferation, indicating that AS1-S may also contribute to the prolonged lifespan of infected cells and thereby increase the probability of acquiring lethal mutations that lead to the progression of lymphoma. This finding may be useful in unveiling novel molecular mechanisms underlying tumorigenesis in BLV-infected cells.

Several factors have been reported to be important determinants related to the pathogenesis of BLV, and integration of the results of the present study with previous insights is expected to contribute to future research for clarifying the mechanism of BLV pathogenesis. As for viral factors, the transcriptional activity defined by interactions between LTRs and the TAX protein has been well characterized (24 – 26). Since transcriptional activity strongly affects BLV replication, it is expected to contribute to the expansion of BLV during the initial infection step. On the other hand, since latent BLV rarely expresses viral proteins, the functions of LTR and TAX cannot completely explain the behavior of the virus during late-stage infection. In addition to viral factors, host-derived factors, such as BoLA-DRB3 polymorphisms (27, 28) and immune exhaustion during BLV infection (29, 30), have also been well characterized. Furthermore, Durkin et al. provided the novel insight that antisense transcripts from the BLV provirus genome are stably expressed during latent infection and might play pivotal roles in the BLV life cycle (11). Rosewick et al. subsequently showed that BLV antisense transcripts produce chimeric transcripts with host mRNA, a critical step in the progression of lymphoma (31). In the present study, we clarified the binding partner of AS1-S RNA and demonstrated that it functioned as a molecular modulator of bovine hnRNPM-RNA interactions, bringing about a new perspective for better understanding the BLV lifecycle.

Interestingly, human and mouse hnRNPM contributes to the innate immune response against pathogens (32, 33). Additionally, other reports have shown that alternative splicing events modulated by hnRNPM were correlated with tumor progression (34, 35). Regarding the former, Cao et al. reported that hnRNPM was translocated from the nucleus to the cytoplasm in response to infection by RNA viruses, resulting in the suppression of innate immune responses by antagonizing RNA sensors (32). West et al. also reported that hnRNPM modulated the splicing of IL6 mRNA and controlled innate immune responses against bacteria (33). In our study, however, no GO terms related to immune responses were significantly enriched in both the RIP-seq and transcriptome analyses (Fig. 1 and 5). Therefore, it remains unknown whether the interaction between AS1-S and bovine hnRNPM affects the immune responses in BLV-infected cells. Hypothetically, since BLV expresses few viral antigens during the latent phase of infection, suppression of immune responses might not be required in latently infected cells, and the interaction of AS1-S and hnRNPM could have another role other than immune suppression.

One of the limitations of this study is that we were not able to perform gain of function and loss of function analyses using bovine B cells. Currently, bovine B cell lines that are free from BLV infection are not available; therefore, it was impossible to perform a gain of function analysis by transfecting AS1-S into bovine B cells. Since all of the experiments in this study used kidney-derived cells, differences related to the cell type could have introduced some biases, for example, the AS1-S RNA expressed in MDBK cells was mainly located in the cytoplasm despite its expression being driven by the 3´LTR promoter (Fig. S1D in the supplemental materials). In addition to the concerns pertaining to the origin of the cells, the low transfection efficiency in bovine cell lines restricts the implementation of certain experiments (36, 37). Since a transient expression strategy was not suitable for our experiments, the antibiotic selection was used to prepare stably expressing cells, a procedure that had the potential to introduce additional biases into the results. In fact, the PCA plot showed that both the MDBK 3´LTR AS1-S and MDBK mock cells exhibited altered transcriptome profiles relative to the parental MDBK cells (Fig. 1A). Moreover, the transcriptome analysis of MDBK cells expressing AS1-S identified significant DEGs, but the obtained GO terms were likely unrelated to BLV pathogenesis (Fig. 1F through H). Thus, these identified GO terms may have been caused by the biases described above. In addition, it should be noted that we attempted to knockdown the expression of AS1-S RNA in BL3.1 cells; however, the siRNAs and antisense oligonucleotides were unable to successfully downregulate the expression of AS1-S RNA, although we successfully knocked down hnRNPM expression using siRNA (Fig. S4 in the supplemental materials). The low transfection efficiency of BL3.1 cells may impede the knockdown of the expression of AS1-S RNA in the nucleus. Taken together, the limitations related to bovine cells are an obstacle to BLV research. Thus, the establishment of novel bovine B cell lines free from BLV infection and the development of new transfection methodologies that are capable of efficiently transfecting bovine cells are required to advance BLV research.

Another limitation of the present study is the number of samples subjected to the high-throughput analyses, such as the transcriptome and RIP-seq analyses. We applied two replicates per group for the transcriptome analysis because Schurch et al. reported that DESeq2 software with a threshold log2 (fold change) =1.0 could detect >70% of the true positive genes from duplicate samples (38). However, this implies that another important gene set was possibly included in the remaining 30% of false negative genes. Although we validated the true positive genes with real-time RT-PCR (Fig. 1E), this limitation should be carefully considered. On the other hand, our RIP-seq results were obtained from *n* = 1 data, and it is possible that the obtained large number of genes (4,607 genes) also included some false positive genes. However, since the RIP-seq results were also validated with real-time RT-PCR (Fig. 6), at least six genes related to “Pathways in cancer,” i.e.*, TGFB2*, *CREBBP*, *EP300*, *FOS*, *JUN*, and *MYC* were certainly affected by the hnRNPM-AS1-S interaction. Regarding the reason why such a large number of mRNAs was immunoprecipitated with hnRNPM, we hypothesized that several RNA-binding proteins other than hnRNPM also co-immunoprecipitated with the hnRNPM-AS1-S complex since the AS1-S RNA probe could bind some host-derived proteins other than hnRNPM (Fig. 2A). Thus, it is possible that mRNAs targeted by the co-immunoprecipitating RNA-binding proteins were also detected, resulting in the large number of genes. Taken together, the complete picture of the effects brought about by the interaction between AS1-S and hnRNPM remains unknown, and further research is needed for clarifying the issue.

There are some outstanding questions that remain to be clarified after this study. For example, although LC-MC successfully identified bovine hnRNPM as the binding partner of AS1-S, the other <20 kDa band could not be identified (Fig. 2A). Thus, there are additional protein factors involved in mediating AS1-S functions, and it is possible that these factors function independently or in cooperation with hnRNPM. Furthermore, since our study mainly focused on the protein-RNA interaction between AS1-S and hnRNPM, we could not rule out the possibility that this interaction inhibited other protein-protein interactions involving hnRNPM. Additionally, although we found that RRM1 and 2 were the regions of hnRNPM required for binding AS1-S RNA (Fig. 4), the biological importance of each RRM was not clarified. Thus, it remains to be elucidated how the RNA-binding properties of these RRMs affect the biological functions of bovine hnRNPM. Since the functions of bovine hnRNPM have not been elucidated at all, future research aiming to clarify the molecular functions of bovine hnRNPM could also provide new insights for BLV research.

It is worth mentioning that the RIP-seq results of BL3.1 cells were drastically different from those of MDBK-derived cells (Fig. S7 in the supplemental materials). Western blotting analysis showed that the expression levels of bovine hnRNPM were similar in BL3.1 and MDBK-derived cells; however, the RIP-seq data showed that the number of mRNAs co-immunoprecipitating with hnRNPM was much greater in BL3.1 cells than in MDBK cells. One hypothesis for this result is that the RNA-binding ability of hnRNPM is more functionally important and stronger in B cells, and therefore, the variety of hnRNPM-interacting RNAs in B cells is more diverse than in other cells. This hypothesis may explain why only B cells undergo tumorigenesis following BLV infection. Another hypothesis is that persistent BLV infection in BL3.1 cells alters the function of bovine hnRNPM more drastically than exogenous AS1-S RNA. To confirm these hypotheses, the development of a novel bovine B cell line is necessary. Regarding the interaction between BLV transcripts and hnRNPM in B cells, knockdown analysis results showed that siRNAs against hnRNPM did not alter the expression of viral proteins (gp51 and p24) in BL3.1 cells (Fig. S4 in the supplemental materials), indicating that hnRNPM might not be important for the transcription of viral sense RNA. However, since our study could not prove the functional knockdown of hnRNPM, such as the loss of splicing machinery, further studies are required to clarify the role of hnRNPM in the BLV lifecycle.

In the present study, we showed that the retroviral lncRNA AS1-S bound to the host hnRNPM and modulated its RNA-binding profile. This novel insight is expected to bring a new perspective to research into retroviral antisense transcripts. Regarding retroviral antisense transcripts, the HBZ gene encoded in human T-cell leukemia virus type 1 (HTLV-1), which also belongs to the genus Deltaretrovirus of the family Retroviridae, is well characterized (39). While HBZ functions as a protein, its RNA form has bimodal functions in HTLV-infected cells (5); HBZ protein suppresses TAX-mediated transcription through the 5´LTR, whereas HBZ RNA promotes cell proliferation and inhibits apoptosis (40). HBZ RNA is involved in the upregulation of the expression of many genes related to the cell cycle, proliferation, and survival by interacting with their promoter sequences (41). Additionally, HBZ RNA directly affects interactions between RNA polymerase and the viral LTR promoter by displacing a transcription factor (42). Thus, diverse roles of HBZ RNA have been reported, and our finding that the retroviral lncRNA, AS1-S, interacts with host hnRNPM might also broaden the understanding of the role of retroviral antisense transcripts in the lifecycle of retroviruses. Evaluation of the similarities and differences between HBZ and AS1-S might reveal important insights relevant to BLV research as well as comparative studies of retroviruses.

## MATERIALS AND METHODS

### Cells

MDBK cells were maintained in Eagle’s medium (Nissui Pharmaceutical, Tokyo, Japan) supplemented with 5% heat-inactivated fetal bovine serum (FBS) (Thermo Fisher Scientific, Waltham, MA, USA), 100 units/mL penicillin, 100 µg/mL streptomycin (Sigma-Aldrich, St. Louis, MO, USA), and 2 mM l-glutamine (Nacalai Tesque, Kyoto, Japan). 293T cells (ATCC CRL-3216) were maintained in Dulbecco’s modified Eagle’s medium (DMEM; Nissui Pharmaceutical) supplemented with 10% FBS along with 100 units/mL penicillin and 100 µg/mL streptomycin. BL3.1 cells (ATCC CRL2306), which are persistently infected with BLV and have been previously characterized (43, 44), were maintained in RPMI1640 GlutaMAX (Thermo Fisher Scientific) supplemented with 10% FBS along with 100 units/mL penicillin and 100 µg/mL streptomycin. Primary bovine lymphocytes were collected from the lymph node of a necropsied BLV-positive calf; the clinical sample was provided by Yamaguchi University.

### Construction of plasmids

We used an expression plasmid encoding BLV AS1-S under the control of the CAG promoter that has been reported previously [pCAG AS1-S (45)]; briefly, 571 bp of AS1-S cDNA was amplified from the total RNA of FLK-BLV cells, which are persistently infected with BLV and express AS1-S RNA (LC164083.1). The obtained amplicon was cloned into the *EcoR*I and *Not*I recognition sites of the expression plasmid pCAG neo (FUJIFILM Wako, Tokyo, Japan) using an In-Fusion HD Cloning Kit (TaKaRa, Shiga, Japan) (Fig. S1A). Because promoter sequences alter the localization of antisense RNA encoded in deltaretroviruses (19), an expression plasmid without the CAG promoter was also constructed; the CAG promoter sequence was removed from pCAG AS1-S by digesting with *Nde*I and *Sal*I (Fig. S1A), and the resultant fragment lacking the CAG promoter was treated with DNA polymerase (Blunting high; TOYOBO, Osaka, Japan) to generate blunt ends, followed by self-ligation using T4 DNA ligase (Ligation high; TOYOBO). The constructed plasmid was designated as p3´LTR AS1-S because the AS1-S RNA was transcribed under the control of core promoter elements and cis-regulatory elements in its internal 3´LTR promoters (11, 46).

To construct a plasmid for synthesizing an AS1-S RNA probe, the AS1-S sequence was obtained from pCAG AS1-S by digesting with *Hin*dIII and *Pst*I. The fragment was subsequently inserted into the pSPT19 plasmid (designated as pSPT19 AS1-S) using the same restriction enzymes.

To construct expression plasmids encoding recombinant hnRNPMs, the complete cDNA sequence of bovine hnRNPM (NM_001191223) was obtained from the total RNA of MDBK cells using the PrimeScript RT Reagent Kit (TaKaRa), followed by PCR using PrimeSTAR Max DNA Polymerase (TaKaRa) with primer no. 1 and 4 (all primers are listed in Table S1 in the supplemental materials). The PCR conditions were as follows: 35 cycles of 98°C for 10 s, 55°C for 5 s, and 72°C for 2 min. The amplicon was then cloned into the *Eco*RI and *Not*I recognition site of the expression plasmid pCAG neo using the HD Cloning Kit (TaKaRa). To construct deletion mutants of bovine hnRNPM, sequence fragments were amplified from the complete hnRNPM cDNA using PrimeSTAR Max DNA Polymerase (TaKaRa) with primer sets no. 1–6; the PCR conditions were the same as those used for amplification of the complete hnRNPM sequence. The resultant fragments were cloned into the *Eco*RI and *Not*I recognition sites of pCAG neo using the HD Cloning Kit (TaKaRa). All recombinant proteins were fused with a his-tag, and expression was confirmed using an anti-his tag antibody.

### Design of small interfering RNAs (siRNAs)

Two pairs of stealth RNAi targeting bovine hnRNPM were designed and purchased from Thermo Fisher Scientific. The paired sequences were sihnRNPM1: 5´-GGCAGUCACUUAAAGACCUGGUUAA-3´ (sense) and 5´-UUAACCAGGUCUUUAAGUGACUGCC-3´ (antisense) and sihnRNPM2: 5´-GAGGUAACAUACGUGGAGCUCUUAA-3´ (sense) and 5´-UUAAGAGCUCCACGUAUGUUACCUC-3´ (antisense). The scrambled sequences 5´-GGCCUCAUUAAGACAUCGGUGAUAA-3´ (sense) and 5´-UUAUCACCGAUGUCUUAAUGAGGCC-3´ (antisense) were used as a control.

### Transfection or nucleofection and establishment of stable cell lines

The constructed plasmids encoding AS1-S were transfected into MDBK cells using the Amaxa Cell Line Nucleofector Kit R (Lonza, Kanagawa, Japan) with the Amaxa Nucleofector II system in accordance with the manufacturer’s instructions. Briefly, 1 µg of plasmid DNA and 1 × 10^6^ cells were mixed with the nucleofector reagent, followed by electroporation using the Amaxa Nucleofector II System with the installed condition program X-001. After nucleofection, the cells were subjected to antibiotic selection using the aminoglycoside G418 (Thermo Fisher Scientific).

293T cells were transfected with the plasmids encoding recombinant bovine hnRNPMs using polyethylenimine (PEI). Briefly, 1 µg of each plasmid was mixed with OPTI-MEM (Thermo Fisher Scientific) containing 10 µL of PEI reagent (2 mg/mL concentration of PEI MAX MW 40,000; Polysciences, Warrington, PA, USA) and then transfected into 293T cells grown to confluency in 6-well plates. Transfected cells were harvested at 72–96 h post-transfection and subjected to subsequent experimentation.

siRNAs targeting bovine hnRNPM were transfected into BL3.1 cells using the Amaxa Cell Line Nucleofector Kit V (Lonza) in accordance with the manufacturer’s instructions. Briefly, 10 µL of 20 µM siRNA and 1 × 10^6^ cells were mixed with the nucleofector reagent, and subsequently subjected to nucleofection using the installed condition program O-017. At 72 h post-nucleofection, cells were harvested and washed with PBS and then subjected to western blotting analysis.

The establishment of stable cell lines was performed in accordance with a previous report (45); briefly, expression plasmids encoding AS1-S (pCAG AS1-S and p3´LTR AS1-S) were transfected into MDBK cells, and the transfected cells were maintained in the presence of 1,000 µg/mL of G418 (Thermo Fisher Scientific). As a control, a stable cell line transfected with an empty vector (pCAG neo) was established in the same manner. The established cells were designated as MDBK CAG AS1-S, MDBK 3´LTR AS1-S, and MDBK mock.

### RNA-protein pull-down assay and LC-MS

An RNA probe was synthesized *in vitro* from pSPT19 AS1-S using T7 RiboMAX Large Scale RNA Production Systems (Promega, Madison, WI, USA). Briefly, approximately 1 µg of the plasmid was mixed with T7 polymerase and incubated at 37°C for 4 h. After transcription, the synthesized RNA was purified and biotin-labeled using the Pierce RNA 3´ End Biotinylation Kit (Thermo Fisher Scientific). The obtained biotin-labeled RNA was used as an RNA probe. Similarly, an RNA probe that is the complement to the AS1-S sequence was synthesized from pSPT19 AS1-S using the SP6 RiboMAX Large Scale RNA Production System (Promega) and used as a control probe. All procedures were performed in accordance with the manufacturers’ instructions.

Pull-down assays were performed using the Pierce Magnetic RNA-Protein Pull-Down Kit (Thermo Fisher Scientific) in accordance with the manufacturer’s instructions. Briefly, 2 µg of the RNA probe was mixed with streptavidin magnetic beads and incubated at 4°C for 1 h, followed by mixing with BL3.1 cell lysates prepared using RIPA buffer (Thermo Fisher Scientific). After incubation at 4°C for 1 h, the beads were washed three times with RIPA buffer, and the RNA-protein complexes were suspended using elution buffer. The samples were subjected to sodium dodecyl sulfate-polyacrylamide gel electrophoresis (SDS-PAGE) and stained using a Silver Stain MS Kit (FUJIFILM Wako). Protein bands that were observed only in the AS1-S RNA probe sample were cut out from the gel and subjected to LC-MS. LC-MS was performed by Japan Proteomics Co., Ltd. (https://www.jproteomics.com/).

### SDS-PAGE and western blotting

Samples were mixed with 4 × Laemmli sample buffer (BIO-RAD, Hercules, CA, USA) containing 200 mM dithiothreitol (DTT) and boiled for 5 min at 95°C. The proteins were separated on 5–20% polyacrylamide gradient gels (ATTO, Tokyo, Japan) and transferred to polyvinylidene difluoride (PVDF) membranes using the iBlot 2 Dry Blotting System (Thermo Fisher Scientific). The membranes were then incubated in 5% skim milk (FUJIFILM Wako) in T-PBS buffer (PBS containing 0.05% Tween 20) at room temperature for 1 h. After the blocking step, the membranes were incubated with primary antibodies [rabbit anti-hnRNPM MAb (ab177957; Abcam, Cambridge, UK), mouse anti-BLV gp51 MAb (BLV2; VMRD, Pullman, WA, USA), mouse anti-BLV p24 MAb (BLV3; VMRD), mouse anti-His tag MAb (D291-3; MBL, Tokyo, Japan), or mouse anti-beta-tubulin MAb (05–661; Merck, Darmstadt, Germany)] in 5% skim milk in T-PBS buffer at room temperature for 1 h. After a washing step, the membranes were incubated with the corresponding peroxidase-conjugated secondary antibodies [goat anti-mouse IgG MAb (ab6789; Abcam) or goat anti-rabbit IgG MAb (82–6120; ZYMED Laboratories, Carlsbad, CA, USA)] in 5% skim milk in T-PBS buffer at room temperature for 1 h. The detected proteins were visualized with Super Signal West Dura Extended Duration Substrate (Thermo Fisher Scientific), and images were obtained using the imaging device FluorChem FC2 (ProteinSimple, Tokyo, Japan).

### RNA immunoprecipitation (RIP) assay

The Magna RIP Kit (Merck) was used to perform the RIP assay in accordance with the manufacturer’s instructions. Briefly, 5 µg of mouse anti-hnRNPM MAb (5-RE36; Santa Cruz, Dallas, TX, USA) and a control antibody (component of the kit) were mixed with magnetic beads and incubated at room temperature for 30 min. Subsequently, the beads were mixed with cell lysates that were prepared using the lysis buffer included in the kit. After incubation at 4°C for 3 h, the beads were washed six times with the wash buffer included in the kit, and the obtained protein-RNA complexes were utilized in subsequent experiments.

### Conventional PCR and real-time PCR

For conventional RT-PCR, total RNA was extracted from cells using the RNeasy Mini Kit (QIAGEN, Tokyo, Japan), followed by RT-PCR using PrimeScript One Step RT-PCR Kit Ver.2 (TaKaRa) with primer nos. 7 and 8 (for detecting full-length AS1-S) or 9 and 10 (for GAPDH). The PCR conditions were as follows: 50°C for 30 min, 94°C for 2 min, followed by 35 cycles of 94°C for 30 s, 60°C for 30 s, and 72°C for 30 s.

RNAs encoding U6 and TYR were used as controls for nuclear and cytoplasmic RNA, respectively, and were quantified by real-time RT-PCR using the One Step TB Green PrimeScript RT-PCR Kit II (Perfect Real Time) (TaKaRa) and QuantStudio 3 Real-Time PCR System (Thermo Fisher Scientific) with primers no. 11 and 12 or 13 and 14. AS1-S RNA in the established MDBK CAG AS1-S and MDBK 3´LTR AS1-S cells were quantified in the same manner using primers no. 15 and 16. For quantification of AS1-S RNA in BL3.1 cells, strand-specific real-time RT-PCR was performed as previously reported (47) to determine the sense and anti-sense transcript levels. Briefly, RNA samples were reverse-transcribed using the Prime Script RT Reagent Kit (TaKaRa) in combination with an AS1-specific tagged primer (no. 17). The resultant reaction mixture was then 10fold diluted and subjected to quantitative PCR using the TB Green Premix Ex Taq II Kit (TaKaRa) in combination with a tag primer (no. 18) and an AS1-specific reverse primer (no. 19). All PCR conditions were in accordance with the manufacturers’ instructions.

For validation of the high-throughput sequencing analyses, such as the transcriptome and RIP-seq analyses, target genes were quantified by real-time RT-PCR using the One Step TB Green PrimeScript RT-PCR Kit II (Perfect Real Time) (TaKaRa) and QuantStudio 3 Real-Time PCR System (Thermo Fisher Scientific) with primers no. 20–41. All PCR conditions were in accordance with the manufacturers’ instructions.

The BLV proviral load was measured as previously described (45). Briefly, a commercial real-time PCR kit for BLV detection (RC202A; TaKaRa) was used to determine the copy numbers of the BLV-pol gene and the bovine RPPH1 gene in DNA samples by multiplex real-time PCR according to the manufacturer’s instructions. The PCR conditions were as follows: 25°C for 10 min and 95°C for 30 s, followed by a two-step procedure for 45 cycles at 95°C for 5 s and 60°C for 30 s. The proviral load data were normalized to the number of BLV-pol gene copies per 100 cells, which was calculated based on the copy number of the bovine RPPH1 gene (two copies per cell).

### Analysis of the subcellular localization of RNAs

Nuclear and cytoplasmic RNAs were separately extracted in accordance with a protocol provided by QIAGEN with slight modifications (https://www.qiagen.com/us/resources/download.aspx?id=1f8ed6e7-2423-4cff-8013-0eb89ead5cb0&lang=en&ver=1: accessed 30 October 2022). Briefly, cell pellets were lysed with RLN buffer (50 mM Tris-HCl, 140 mM NaCl, 1.5 mM MgCl_2_, 0.5% NP40), and nuclear and cytoplasmic fractions were separated by centrifugation, followed by RNA extraction using NucleoSpin RNA Plus (TaKaRa).

### RNA-seq and transcriptome analysis

For RNA-seq-based transcriptome analysis, total RNA was extracted from MDBK 3´LTR AS1-S, MDBK mock, and parental MDBK cells using NucleoSpin RNA Plus (TaKaRa) (*n* = 2). Construction of RNA-seq libraries and sequencing were performed by Macrogen Japan Co., Ltd. (https://www.macrogen-japan.co.jp/); sequencing libraries were prepared using the TruSeq stranded mRNA LT Sample Prep Kit (Illumina, San Diego, CA, USA), and next-generation sequencing analysis was conducted using NovaSeq 6000 (100 bp paired-end reads, approximately 4-Gbp total read bases per sample). The obtained data were trimmed using Trimmomatic (ver. 0.36), then mapped to the reference genome (Bos taurus genome: ARS-UCD1.2) using HISAT2 (ver. 2.0.4), followed by transcript assembly and quantification using StringTie (ver. 2.1.1). After the assembly step, genes with read counts of <10 in at least one sample were removed. PCA was performed using prcomp (Stats package in R ver. 4.3.0), and differential expression analysis was carried out using DESeq2 (ver. 1.40.1), followed by GO analysis using clusterProfiler [(48), ver. 4.8.1]. DEGs were determined between the MDBK 3´LTR AS1-S cells (*n* = 2) and the MDBK mock and parental cells (*n* = 4) to identify specific changes caused by the expression of AS1-S. To obtain supplemental data, multiple comparisons between the three groups were also performed. Significant DEGs were defined based on the following criteria: |fold change| ≥2, exactTest adjusted *P*-value <0.05. For the analysis of the mRNA distribution, nuclear and cytoplasmic RNA were subjected to RNA-seq, followed by data manipulation as described above. The results of GO analysis are shown using the simplify option in clusterProfiler.

### RIP-seq analysis

RNA obtained from the RIP assay sample was purified according to the manufacturer’s instructions, followed by library construction and RNA-seq analysis (*n* = 1). The construction of RNA-seq libraries and sequencing were performed by Macrogen Japan Co., Ltd. The sequence libraries were prepared using the SMARTer Stranded RNA-Seq Kit (Illumina), and next-generation sequencing analysis was conducted using NovaSeq 6000 (100 bp paired-end reads, approximately 4-Gbp total read bases per sample). The obtained data were filtered using prinseq (ver. 0.20.4) to remove abnormal reads with a GC content <20 and >70. Subsequently, the obtained reads were trimmed using Trimmomatic (ver. 0.36) and mapped to the reference genome (Bos taurus genome: ARS-UCD1.2) using HISAT2 (ver. 2.0.4), followed by transcript assembly and quantification using StringTie (ver. 2.1.1). After assembly, genes with read counts of 0 in all samples were removed, and the read counts for each gene were compared between the samples from the RIP assay with the anti-hnRNPM MAb and those with the control Ab in each cell line. Genes that met the criteria described below were defined as hnRNPM-binding RNAs: read count >10 and hnRNPM/control ratio >2.0. The resultant hnRNPM-binding RNA lists were compared between MDBK 3´LTR AS1-S and MDBK mock cells, and genes that were observed in only MDBK 3´LTR AS1-S cells were subjected to GO analysis using clusterProfiler (ver. 4.8.1). The results of GO analysis are shown using the simplify option in clusterProfiler.

## Data Availability

The primers used in this study are listed in Table S1 in the supplemental materials. The FASTQ data from this study were deposited in the DDBJ database under submission no. DRR444704–444720 and DRR446101. All supplemental information is provided in the supplemental materials.

## SUPPLEMENTAL MATERIAL

Fig. S1 to S7 spectrum.00855-23-s0001.pdf Supplemental figures.

Tables S1 to S13 spectrum.00855-23-s0002.xlsx Supplemental tables.

ASM does not own the copyrights to Supplemental Material that may be linked to, or accessed through, an article. The authors have granted ASM a non-exclusive, world-wide license to publish the Supplemental Material files. Please contact the corresponding author directly for reuse.
