# Supplementary material for: The bovine leukemia virus-derived long non-coding RNA AS1-S binds to bovine hnRNPM and alters the interaction between hnRNPM and host mRNAs
Source: Microbiol Spectr. 2023 Sep 6;11(5):e00855-23. doi: 10.1128/spectrum.00855-23 (PMC10581181; doi:10.1128/spectrum.00855-23)
Supplement: Fig. S1 to S7 — Supplemental figures. [file spectrum.00855-23-s0001.pdf]

Fig. S1 (related to Fig. 1)

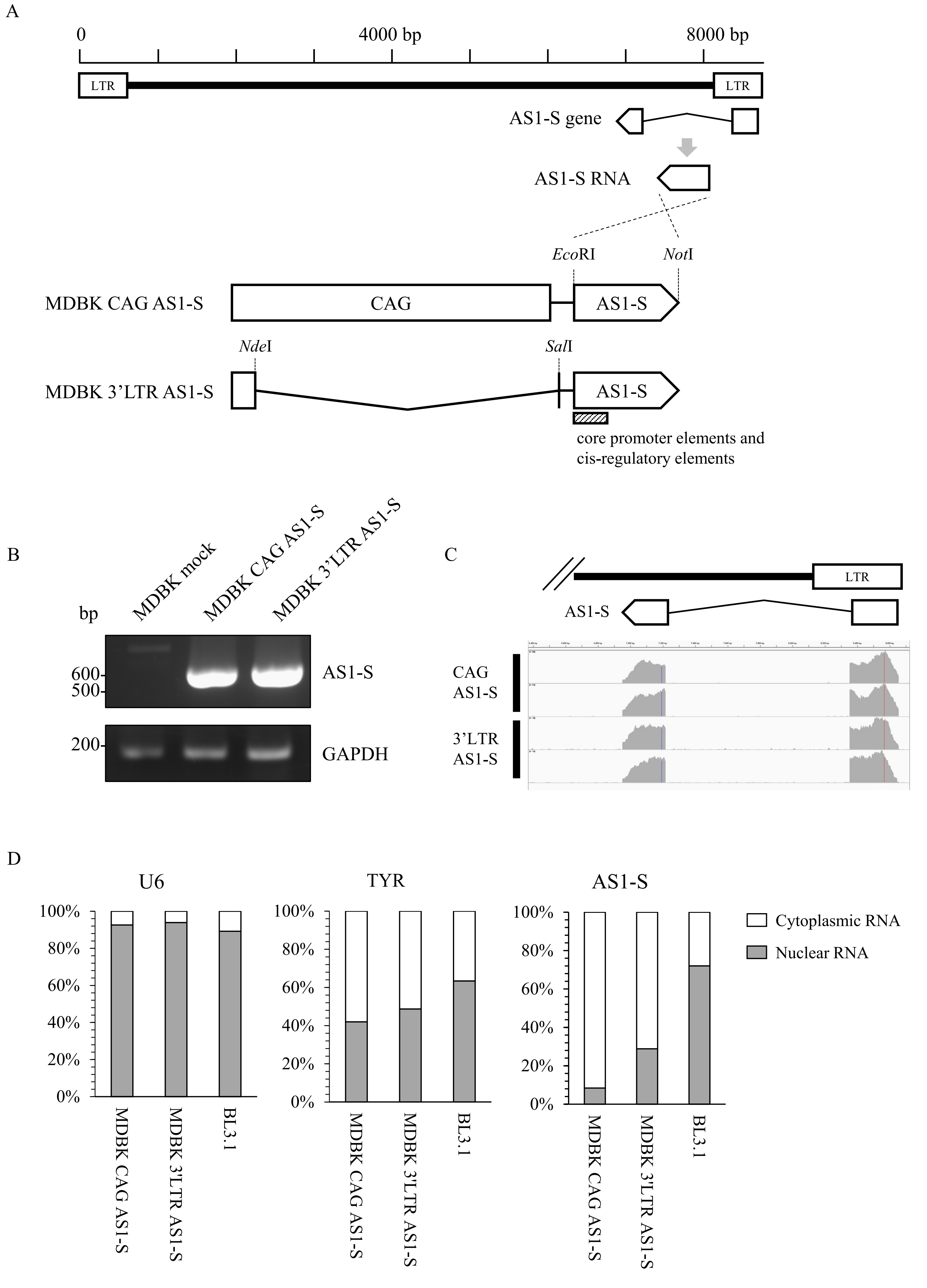

Fig. S1

(A) Schematic diagrams of the AS1-S expression plasmids. CAG: CAG promoter, AS1-S: cDNA sequence of BLV AS1-S. The core promoter elements and cis-regulatory elements include the motif ten element (MTE), downstream promoter element (DPE), TFIIB-recognition element (BRE), interferon regulatory factor (IRF), and E-Box 4 (46).

(B) Results of the RT-PCR amplification of whole AS1-S RNA and the housekeeping gene GAPDH. Total RNA was extracted from transfected cells and subsequently subjected to conventional RT-PCR.

(C) Mapping results of the RNA-seq reads used for the transcriptome analysis in Fig. 1. The FLK-BLV genome (LC164083) was used as the reference genome, and the region encoding AS1-S is enlarged. The Y axis shows the number of mapped reads in each sample. The mapping results were visualized using the Integrative Genomics Viewer (IGV).

(D) Subcellular localization of U6, TYR and AS1-S RNAs in transfected cells. Nuclear (gray) and cytoplasmic (white) RNA were separately extracted from the cells, followed by real-time RT-PCR. The relative amounts of cytoplasmic and nuclear RNAs (n = 3) are shown as percentages.

Fig. S2 (related to Fig. 1)

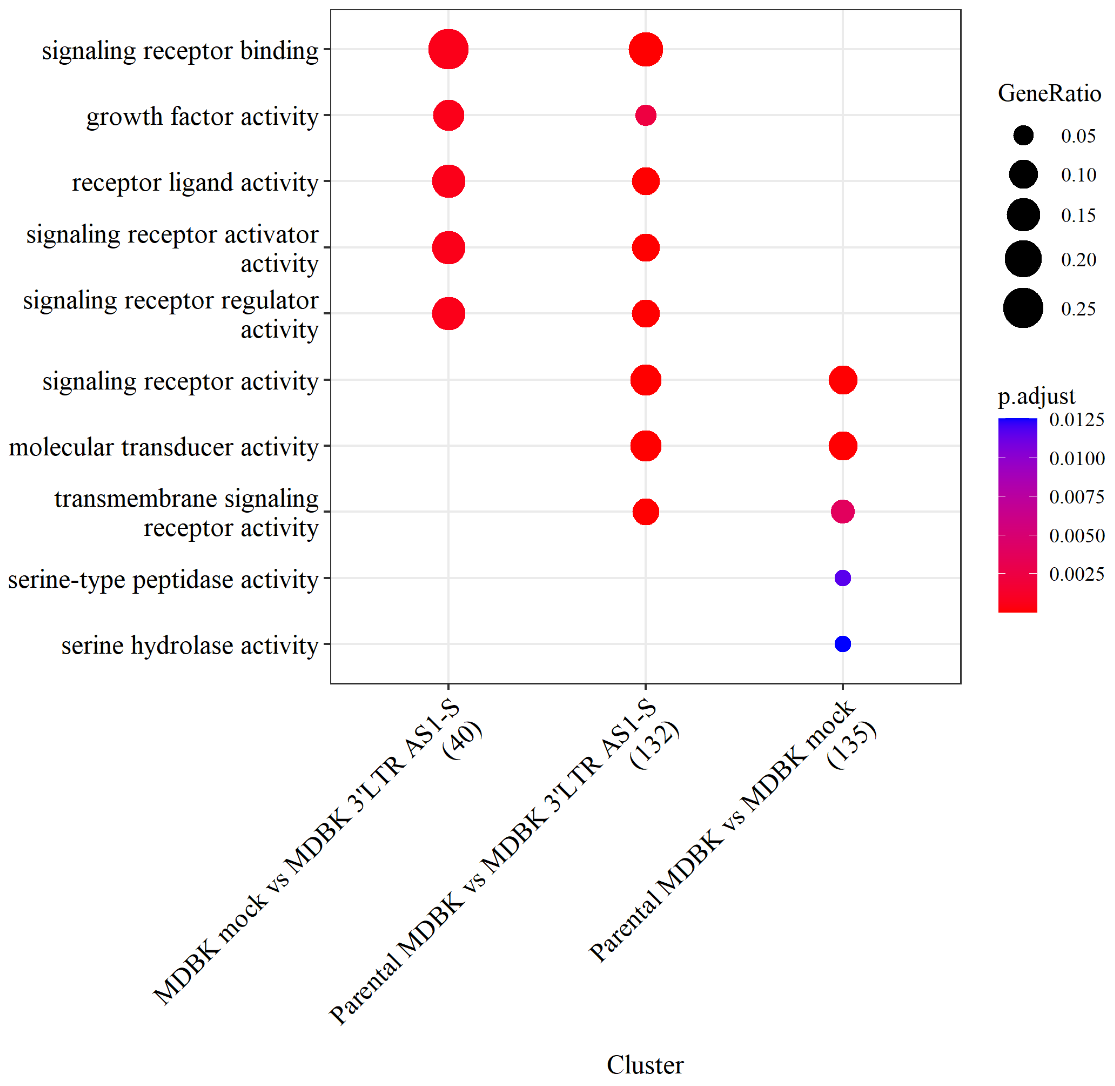

Fig. S2

Gene ontology (GO) analysis of significant differentially expressed genes (DEGs) from multiple comparisons. The significant DEGs identified in each group were subjected to GO analysis, and the results are shown using the compareCluster function.

Fig. S3 (related to Fig. 2)

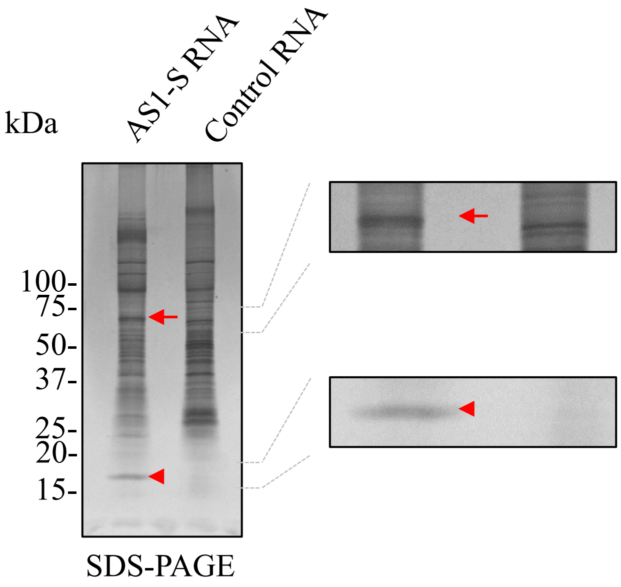

Fig. S3

Results of the pull-down assay using RNA probes with a high concentration of BL3.1 cell lysate. Biotinylated AS1-S or control RNA probes were mixed with the lysate, and pulled-down samples were subjected to SDS-PAGE (silver staining). The red arrow shows the band that was predicted to be bovine hnRNPM by LC-MS. The arrowhead shows the band that is specific for the AS1-S RNA probe, but did not produce a significant result with LC-MS.

Fig. S4

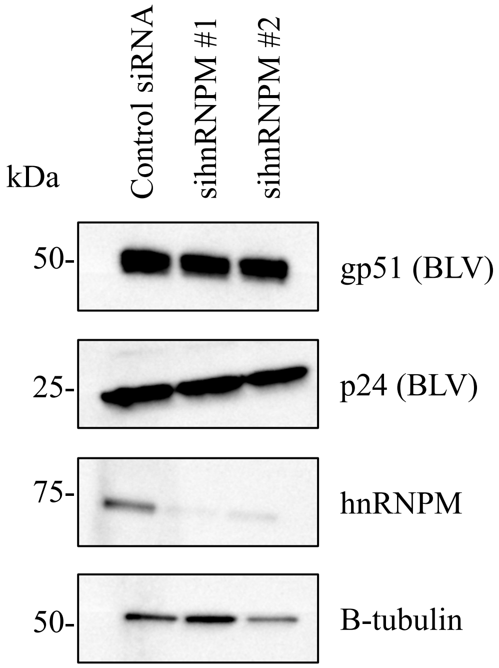

Fig. S4

Western blotting analysis of BL3.1 cells transfected with siRNAs. Two siRNAs against bovine hnRNPM and a scrambled control were transfected into BL3.1 cells and the expression of each protein was visualized using monoclonal antibodies.

Fig. S5 (related to Fig. 5)

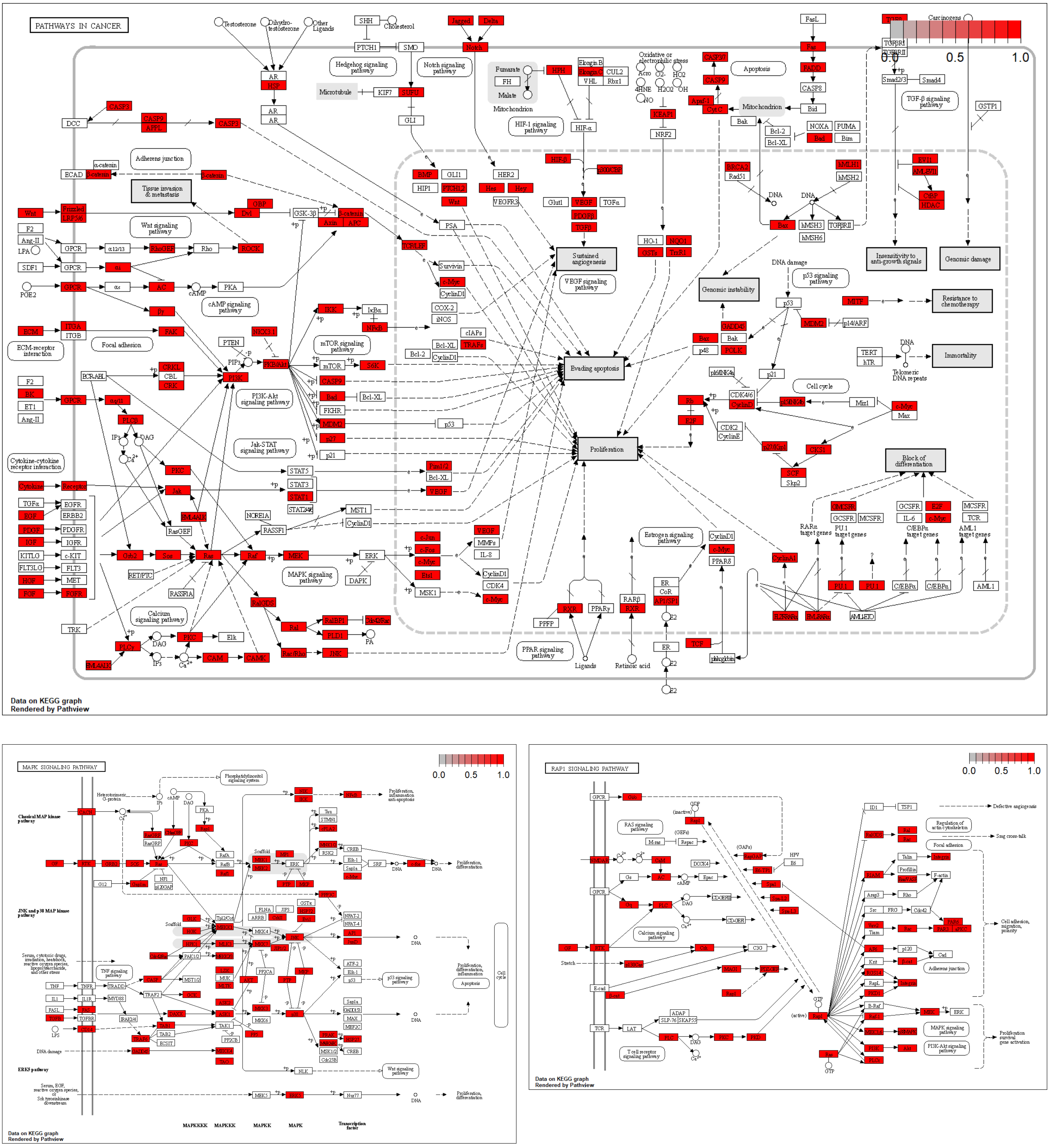

Fig. S5

Diagrams of the KEGG pathways “bta05200 (Pathways in cancer)”, “bta04010 (MAPK signaling pathway)”, and “bta04015 (Rap1 signaling pathway)”. Red indicates genes for which hnRNPM-binding RNAs were detected in only MDBK 3´ LTR AS1-S cells as identified by RIP-seq.

Fig. S6

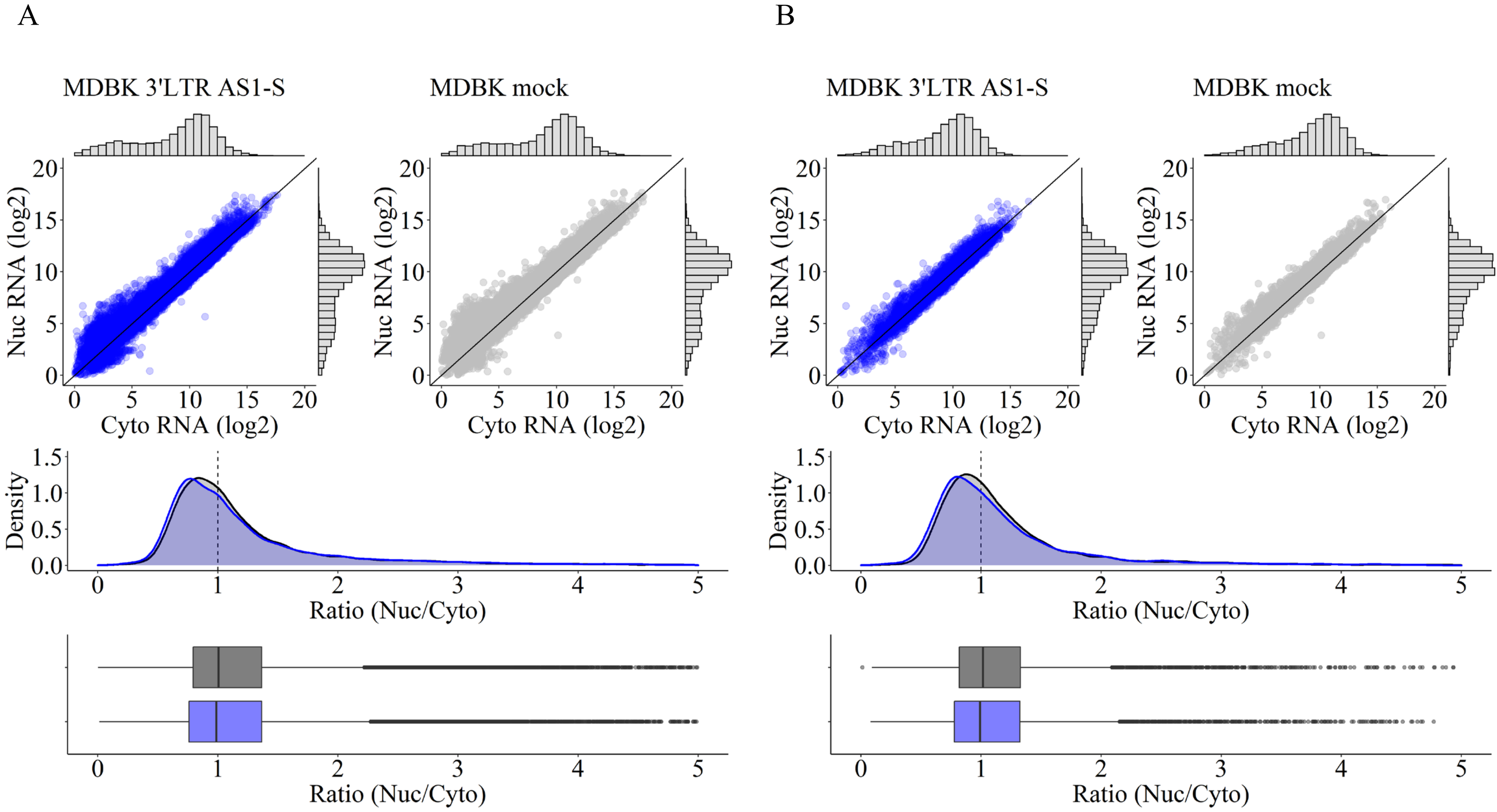

Fig. S6

(A) Scatter plots showing the read counts of nuclear (Nuc) and cytoplasmic (Cyto) RNAs extracted from MDBK 3´LTR AS1-S and MDBK mock cells. The read counts were normalized using DEseq2 prior to analysis. The X and Y axes show the read counts of cytoplasmic (Cyto) and nuclear (Nuc) RNAs, respectively. The bottom graph shows a density plot and box plot of the nuclear/cytoplasmic RNA ratio in MDBK 3´LTR AS1-S (blue) and MDBK mock (black) cells. The X axis shows the calculated ratio of the nuclear and cytoplasmic RNAs. The vertical lines indicate a Nuc/Cyto RNA ratio = 1.0.

(B) The mRNAs of the 4607 genes in Fig. 5C were extracted from the results shown in panel (A).

Fig. S7

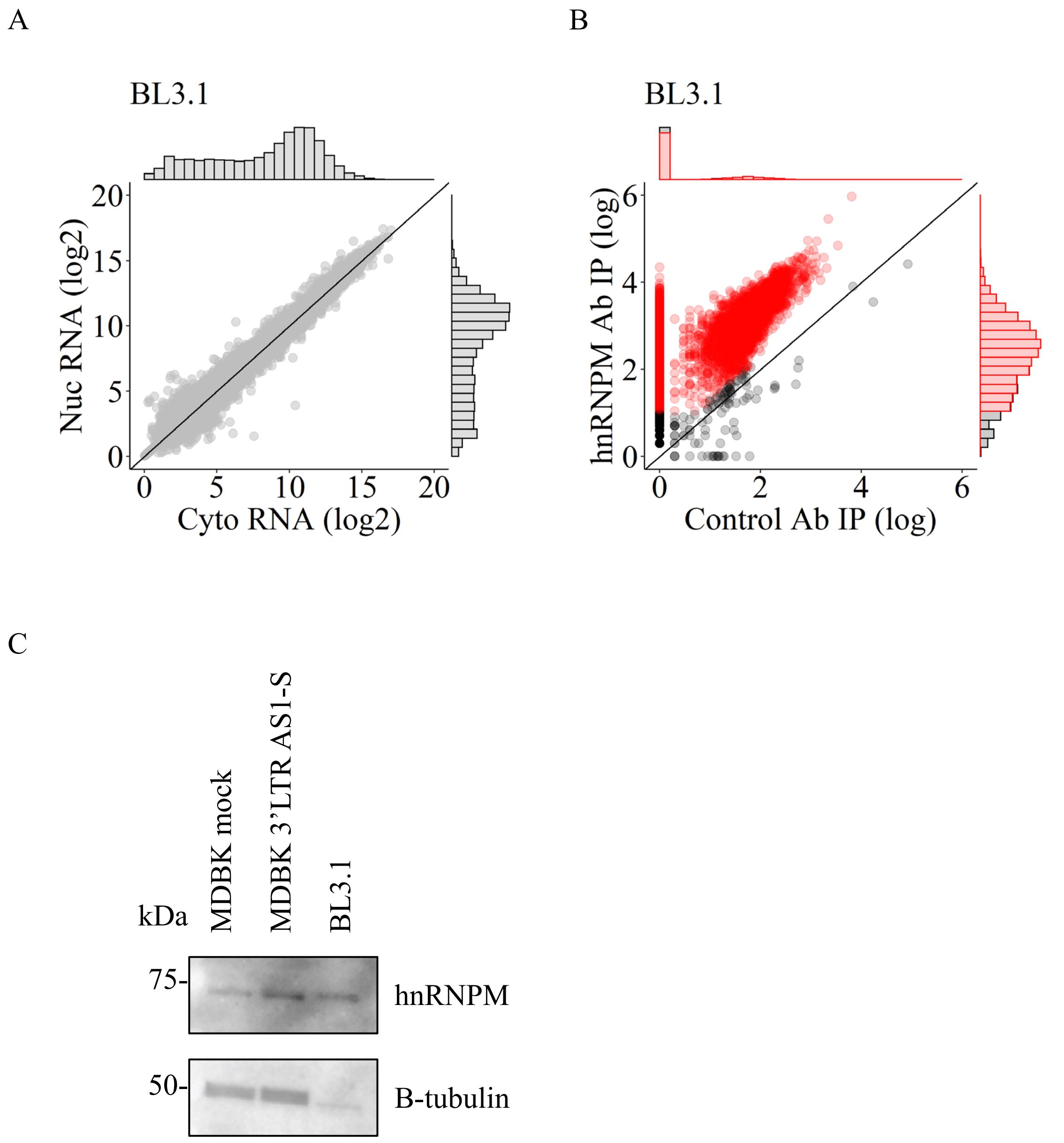

Fig. S7

(A) Scatter plot showing the read counts of nuclear (Nuc) and cytoplasmic (Cyto) RNAs extracted from BL3.1 cells. The read counts were normalized using DEseq2 prior to analysis. The X and Y axes show the read counts of the cytoplasmic (Cyto) and nuclear (Nuc) RNAs, respectively.

(B) Scatter plot showing the sequencing reads obtained by RNA immunoprecipitation using BL3.1 cells. The X and Y axes show the read counts obtained by RIP-seq with the control MAb and anti-hnRNPM MAb, respectively. Genes that met the following criteria were defined as hnRNPM-binding RNAs and are shown as red dots: a read count > 10, hnRNPM/control ratio > 2.0.

(C) Western blot analysis comparing the expression levels of hnRNPM in BL3.1, MDBK 3 ´ LTR AS1-S and MDBK mock cells. 1 × 106 cells were subjected to the analysis.
